# Refractory Cervical Necrotizing Fasciitis Successfully Controlled by Submandibular Gland Excision: A Case Report

**DOI:** 10.1002/ccr3.72564

**Published:** 2026-04-20

**Authors:** Hiroyuki Makihara, Shoichiro Kitajima, Sanae Maeda, Susumu Mizuno

**Affiliations:** ^1^ Department of Oral and Maxillofacial Surgery Daiyukai General Hospital Aichi Japan; ^2^ Department of Oral and Maxillofacial Surgery Sakura General Hospital Aichi Japan

**Keywords:** case report, cervical necrotizing fasciitis, debridement, necrotizing fasciitis, submandibular gland

## Abstract

Persistent cervical necrotizing fasciitis may indicate an unrecognized infectious reservoir. In refractory cases, excision of a suspected salivary gland may be required to achieve definitive infection control when repeated debridement fails.

## 1 Introduction

Necrotizing fasciitis (NF) is a rare but fulminant soft tissue infection characterized by rapidly progressive necrosis of the fascia and subcutaneous tissue, associated with high mortality and morbidity [1, 2]. The condition was first described by Jones in 1871 [3], and various terms such as hospital gangrene and hemolytic streptococcal gangrene have historically been used [4]. Wilson later introduced the term NF in 1952, which remains the most accurate descriptor of the disease [5].

The reported incidence of NF ranges from 0.3 to 5 per 100,000 population, with mortality rates between 30% and 43% [6, 7, 8, 9]. Although the disease most commonly affects the extremities, abdominal wall, and perineum, involvement of the cervical region is relatively rare [10]. Cervical NF typically originates from odontogenic, pharyngeal, or iatrogenic infections and can rapidly spread along superficial and deep fascial planes, leading to airway compromise and systemic sepsis [10, 11].

Computed tomography (CT) plays a critical role in early diagnosis, particularly through the detection of subcutaneous gas and deep space involvement [12]. In addition, laboratory‐based tools such as the Laboratory Risk Indicator for Necrotizing Fasciitis (LRINEC) score and serum procalcitonin levels have been proposed to aid early diagnosis and risk stratification [13, 14, 15, 16, 17, 18, 19, 20, 21]. Despite these diagnostic advances, prompt surgical debridement combined with appropriate antimicrobial therapy remains the cornerstone of treatment [22]. In most cases, extensive debridement is sufficient to control disease progression. However, the optimal management strategy remains unclear when necrosis persists despite repeated surgical intervention. Here, we report a case of cervical NF in which adequate debridement and antimicrobial therapy failed to control disease progression, and excision of the submandibular gland resulted in definitive infection control.

## 2 Case History/Examination

A 68‐year‐old man was admitted to the outpatient department of Daiyukai General Hospital in January 2017 with complaints of progressive swelling in the left mandibular region and odynophagia.

The patient had a medical history of chronic renal failure and was receiving nicorandil, lansoprazole, carvedilol, rebamipide, precipitated calcium carbonate, and phosphate binders. He had no history of smoking, alcohol consumption, or previous inflammatory disease of the head and neck region.

On presentation, his vital signs were as follows: heart rate 116 beats/min, blood pressure 151/76 mmHg, body temperature 37.1°C, and oxygen saturation 99% on room air. His Glasgow Coma Scale score was E4V5M6 (15 points). Laboratory investigations revealed a white blood cell count of 12,300/μL (neutrophils 92.4%), hemoglobin 15.6 g/dL, platelet count 12.5 × 10^4^/μL, and C‐reactive protein 20.2 mg/dL. Renal function was markedly impaired, with a serum creatinine level of 8.88 mg/dL and an estimated glomerular filtration rate of 5.3 mL/min/1.73 m^2^. Serum sodium was 132 mEq/L, potassium 5.9 mEq/L, and glucose 157 mg/dL. The serum procalcitonin level was markedly elevated at 19.21 ng/mL.

**FIGURE 1:**
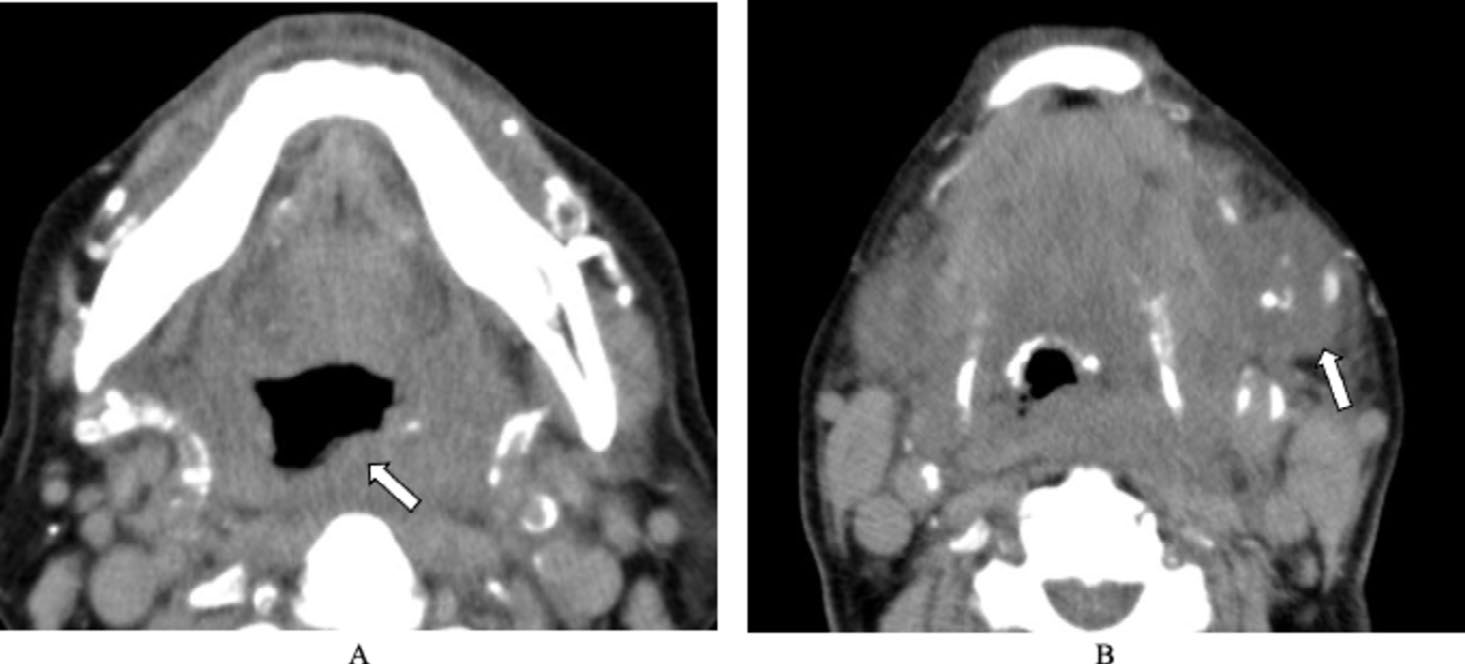
Contrast‐enhanced CT images on the first hospital day. CT images showing airway obstruction due to swelling of the left pharynx (A, arrow) and the left submandibular gland (B, arrow).

Based on these laboratory findings, the patient's LRINEC score was calculated as eight points (Table 1), which is highly suggestive of NF. Contrast‐enhanced CT demonstrated marked swelling of the left submandibular gland and left pharyngeal wall, causing compression of the upper airway, without evidence of a neoplastic lesion (Figure 1). Based on these findings, cervical NF with impending upper airway obstruction was strongly suspected, with left‐sided sialadenitis and pharyngitis considered possible sources of infection.

**TABLE 1:** The LRINEC score.

Variables		Score
CRP (mg/dL)		
< 15		0
≥ 15		4
WBC (/μL)		
< 15,000		0
15,000–25,000		1
> 25,000		2
Hb (g/dL)		
> 13.5		0
11.0–13.5		1
< 11.0		2
Na (mEq/L)		
≥ 135		0
< 135		2
Cre (mg/dL)		
≤ 1.59		0
> 1.59		2
Glu (mg/dL)		
≤ 180		0
> 180		1

*Note:* A LRINEC score of 6 or more is suspicious for necrotizing fasciitis, and a score of 8 or more is highly suggestive of necrotizing fasciitis.

## 3 Differential Diagnosis, Investigations, and Treatment

Several hours after the initial consultation, the patient developed cyanosis. Oxygen therapy was initiated at 8 L/min; however, oxygen saturation deteriorated to 64%, necessitating emergent endotracheal intubation. The patient was transferred to the intensive care unit and started on broad‐spectrum antimicrobial therapy.

**FIGURE 2:**
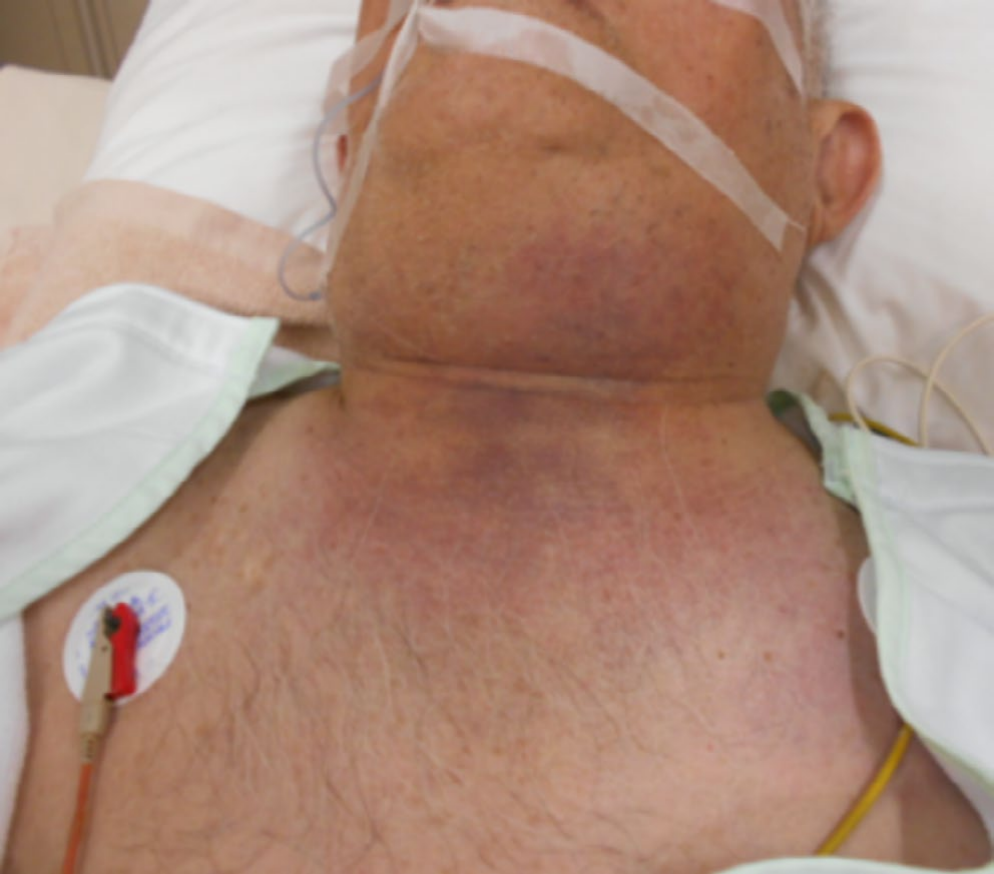
On the morning of the first hospital day. A dark red discoloration was observed extending from the mandible to the neck.

**FIGURE 3:**
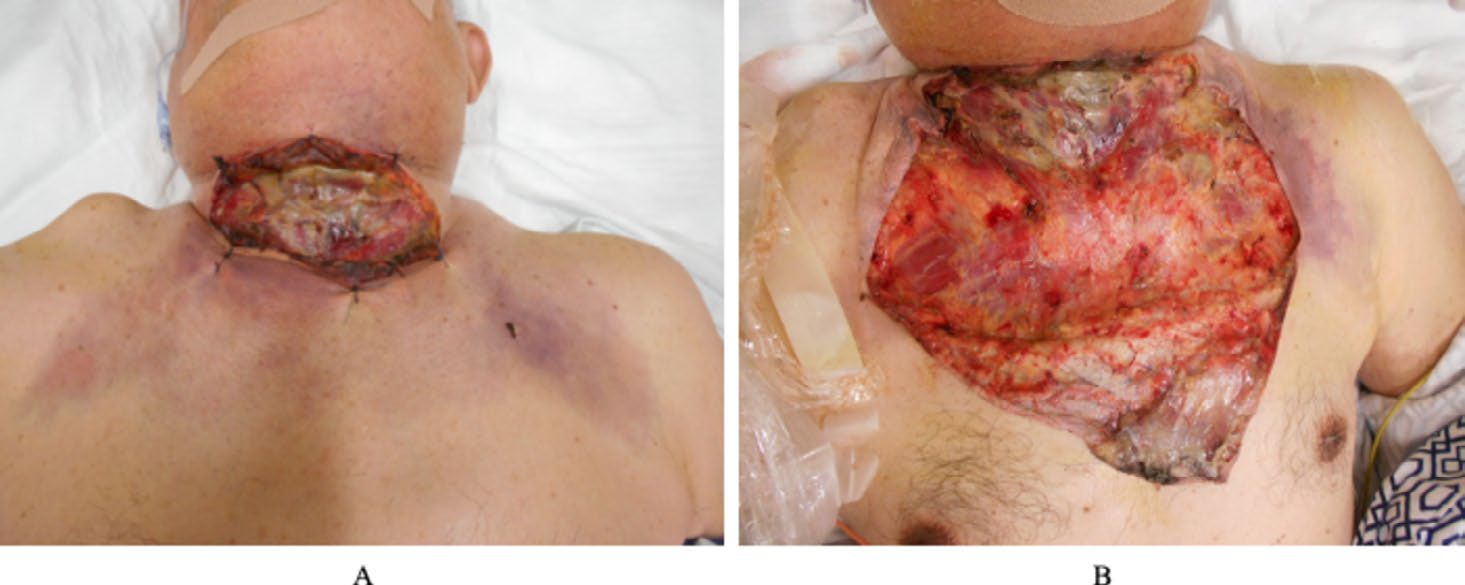
On the evening of the first hospital day. As the dark red discoloration extended to the upper chest (A), additional debridement was performed (B).

On the morning of the first hospital day, rapid progression of skin discoloration was observed, with a dark red change extending from the mandible to the cervical region (Figure 2). A diagnosis of cervical NF was established, and urgent surgical debridement was performed. Despite this intervention, the area of necrosis extended to the upper chest by the evening of the same day, requiring repeat surgical debridement (Figure 3).

**FIGURE 4:**
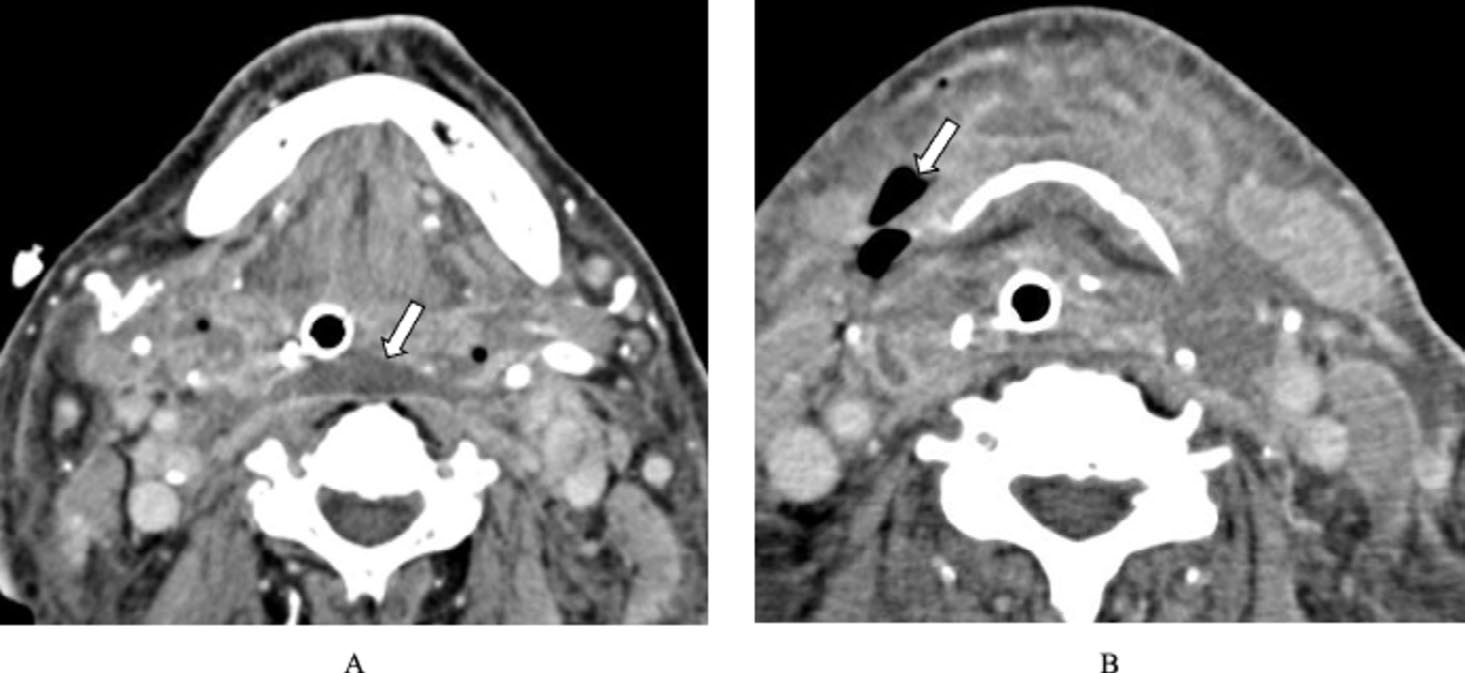
Contrast‐enhanced CT images on hospital Day 12. An abscess extending from the left submandibular gland to the anterior surface of the cervical vertebrae is observed (A, arrow), along with air‐containing lesions within the soft tissue (B, arrow).

**FIGURE 5:**
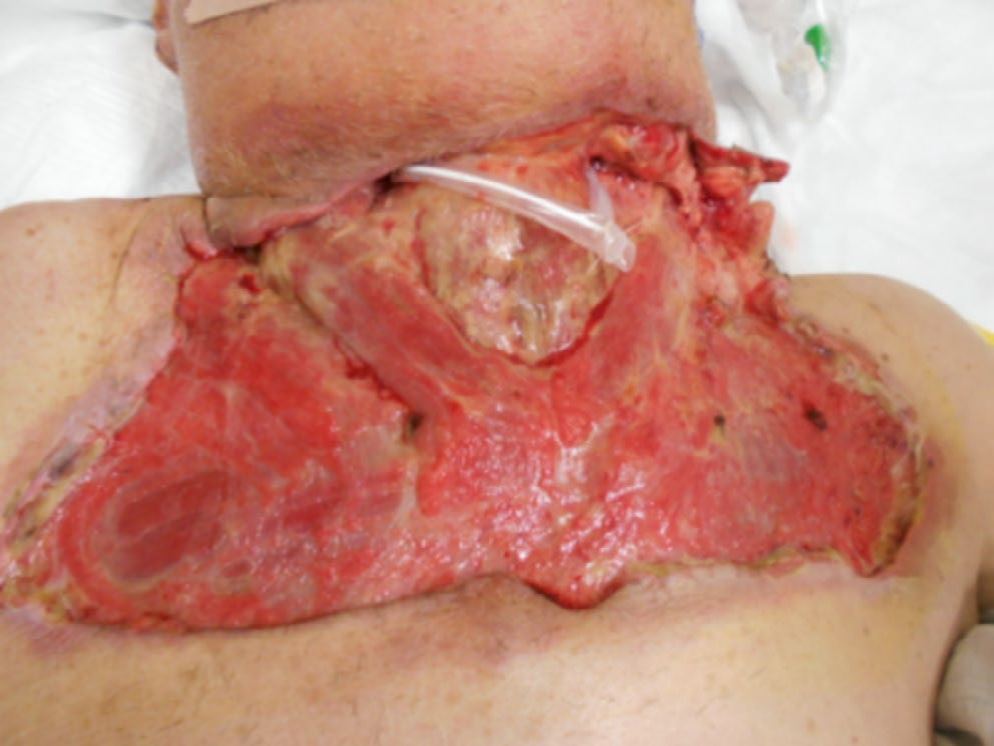
Incisional drainage after debridement. A drainage tube was placed in the dorsal laryngeal region.

On hospital day 4, *Streptococcus pyogenes* was identified from pus cultures. Based on microbiological sensitivity testing and the patient's renal dysfunction with hyperkalemia, antimicrobial therapy was adjusted to sulbactam/ampicillin (3 g/day) and daptomycin (525 mg/day), with dose modification according to renal function. Despite repeated debridement and antimicrobial treatment, contrast‐enhanced CT on hospital Day 12 demonstrated a persistent abscess extending from the left submandibular gland to the anterior surface of the cervical vertebrae, accompanied by gas formation within the soft tissue (Figure 4). Blunt dissection was performed bilaterally along the anterior cervical muscles and caudally from the submandibular gland to the vertebral surface. Necrotic tissue and cloudy fluid were encountered, and a drainage tube was placed in the dorsal laryngeal region (Figure 5).

**FIGURE 6:**
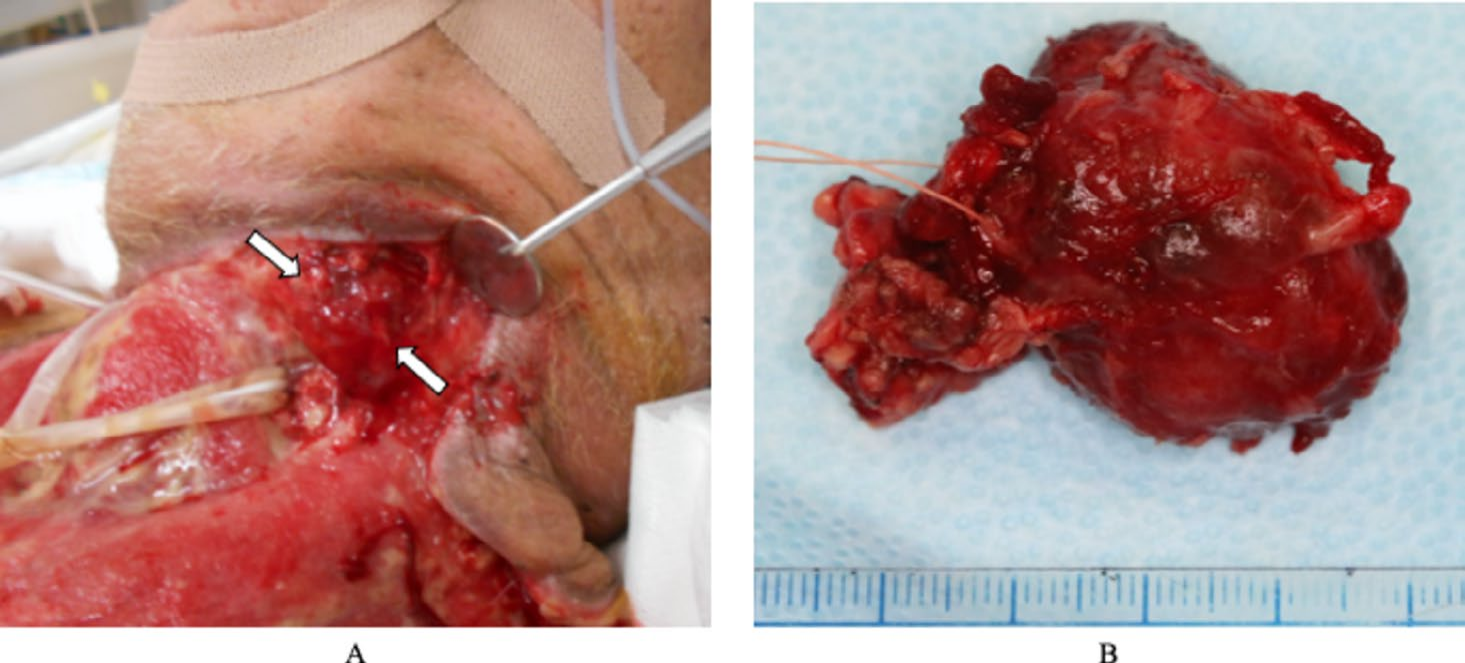
Resected salivary gland tissue. (A) The left submandibular gland showing marked swelling and dark red discoloration (arrow). (B) The excised submandibular gland with surrounding tissue.

Although transient laboratory improvement was observed following repeated surgical interventions, the necrotic process continued to progress. Given the persistent inflammation localized around the left submandibular region, the gland was suspected to represent a residual infectious reservoir. Consequently, on hospital Day 19, a left‐sided submandibular gland excision was performed (Figure 6).

**FIGURE 7:**
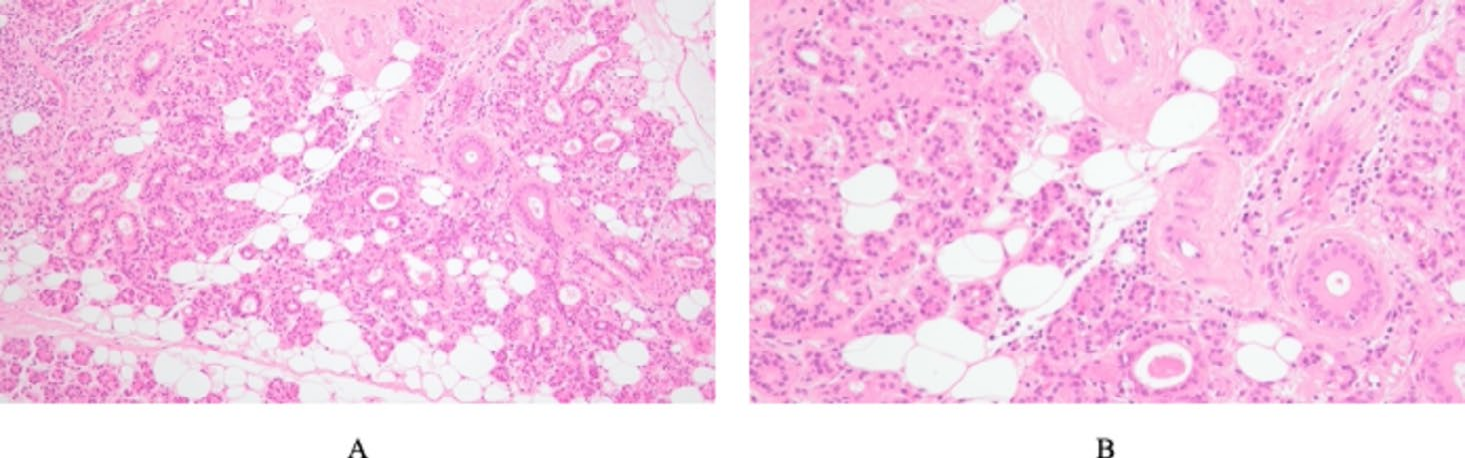
Histopathological findings. (A) Low‐power view demonstrates preserved salivary gland architecture with inflammatory cell infiltration predominantly involving the capsule and surrounding stromal tissue (hematoxylin and eosin stain, ×100). (B) High‐power view shows lymphocyte‐predominant inflammatory infiltration in the pericapsular and stromal areas without evidence of glandular parenchymal destruction, abscess formation, or active infection within the salivary gland tissue (hematoxylin and eosin stain, ×200).

Gross examination revealed necrosis of the glandular capsule, while the glandular parenchyma appeared relatively preserved. Histopathological evaluation demonstrated inflammatory cell infiltration in the capsule and surrounding stromal tissue; however, no obvious evidence of active infection was identified within the salivary gland parenchyma (Figure 7).

Following submandibular gland excision, inflammatory markers gradually improved, and serum procalcitonin levels became negative by hospital Day 25. No further progression of necrotic lesions was observed. On hospital Day 41, reconstruction was performed using split‐thickness skin grafting in combination with the pectoralis major muscle.

## 4 Conclusion and Results (Outcome and Follow‐Up)

**FIGURE 8:**
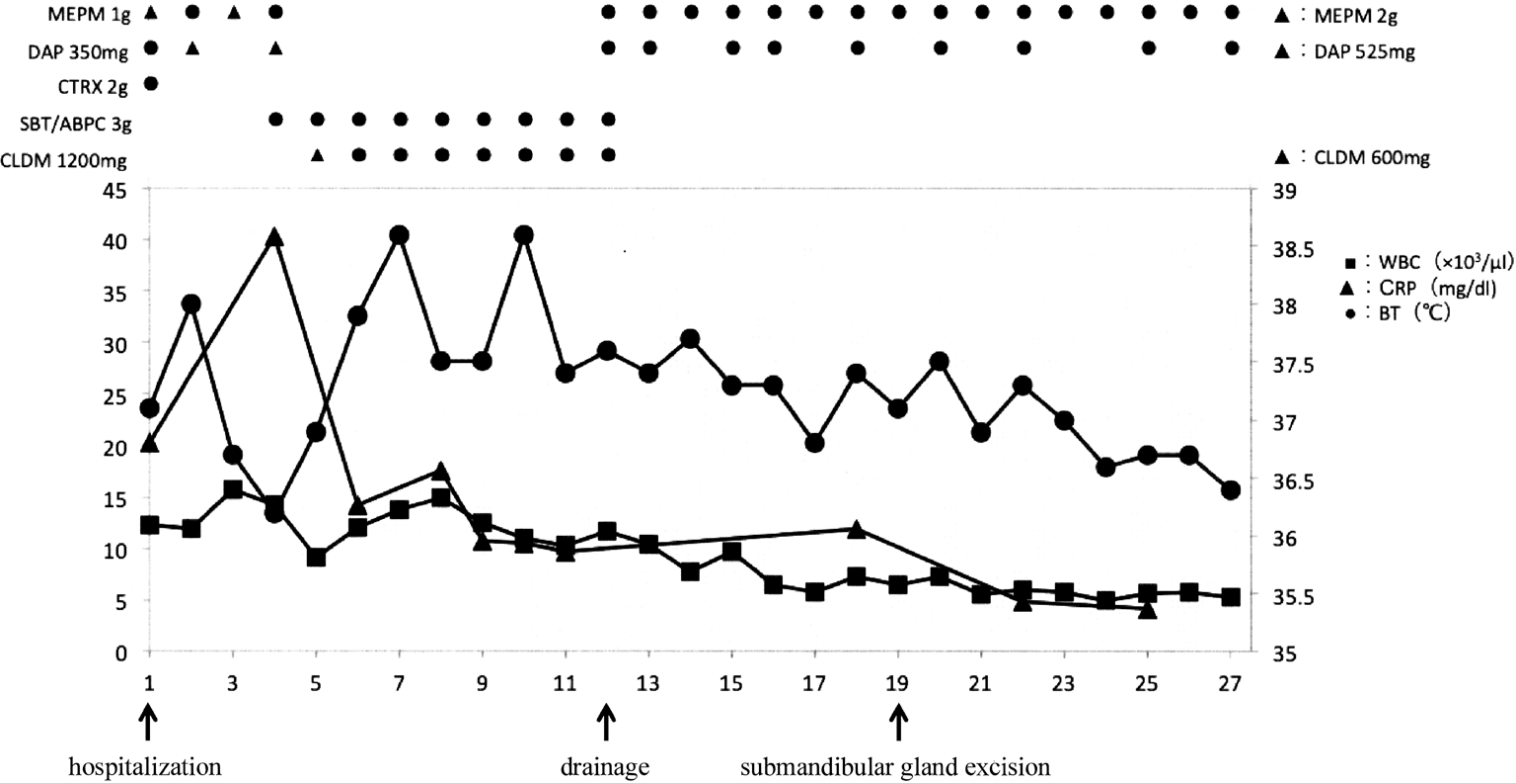
Course and treatment.

The patient was discharged after 69 days of hospitalization and has remained well without recurrence during follow‐up (Figure 8).

## 5 Discussion

Cervical NF is a rapidly progressive infection of the cervical fascia and subcutaneous tissue that can spread along fascial planes within hours. Cervical NF accounts for approximately 2.6% of all head and neck infections [23], and its reported mortality rate ranges from 13% to 19% [24, 25]. The mean age of patients with cervical NF is 49.1 years, with a male predominance (64.23%) [25]. The most common initiating factors are odontogenic infections (47.04%), followed by pharyngolaryngeal (28.34%) and tonsillar or peritonsillar infections (6.07%) [25]. In contrast, tonsillar, salivary gland, otogenic, and dermatologic infections are relatively rare causes [26].

Known risk factors for NF include diabetes mellitus, alcoholism, immunosuppression, obesity, advanced age, chronic renal failure, impaired leukocyte function, and malignancy. In the present case, the patient had chronic renal failure as a significant risk factor and developed nonodontogenic cervical NF.

NF is classified into four types: Type 1 (70%–80%), Type 2 (20%–30%), Type 3 (more common in Asia), and Type 4 (fungal infection) [14]. Type 1 represents polymicrobial infections involving aerobic and anaerobic organisms, whereas Type 2 is caused by a single organism, most commonly group A *Streptococcus* [1, 27]. Reported aerobic pathogens include *Streptococcus* (61.22%) and *Staphylococcus* species (18.09%), while anaerobic organisms such as *Peptostreptococcus* and *Prevotella* account for approximately 10.87% [25].

In our case, no obvious odontogenic infection was identified. However, swelling of the pharynx was noted at the initial visit, and *S. pyogenes* was isolated on bacterial culture. These findings suggest that a pharyngeal infection may have acted as the primary trigger, and the case was therefore categorized as Type 2 NF.

Early surgical debridement is a critical factor associated with improved survival in NF [28, 29]. Previous studies have demonstrated that surgical intervention within 24 h significantly improves survival, and intervention within 6 h further enhances outcomes [24, 30, 31]. Therefore, early recognition of NF is essential to allow prompt and aggressive multidisciplinary treatment.

The LRINEC score, which is based on six routine biochemical and hematological parameters, has been proposed as an adjunctive tool for early diagnosis [18, 22]. A LRINEC score of 6 or more is considered suspicious for NF, and a score of 8 or more is highly suggestive [22]. Procalcitonin, a peptide precursor of calcitonin involved in calcium homeostasis, is another useful biomarker of invasive bacterial infection. When a cutoff value of 5.88 ng/mL is applied, serum procalcitonin reportedly shows both sensitivity and specificity of 100% for differentiating NF from cellulitis [32]. Other diagnostic tools, including the necrotizing soft tissue infection assessment score (NAS) and the SIARI score, have also been advocated [33, 34].

In the present case, both the LRINEC score and elevated serum procalcitonin levels suggested progression to NF. On the morning of the first admission day, rapidly expanding dark red skin discoloration extending from the mandible to the neck was observed, leading to the diagnosis of NF. The combined use of the LRINEC score and serum procalcitonin enabled early recognition and timely surgical debridement, which was crucial for patient survival.

The Infectious Diseases Society of America recommends initial treatment with a combination of broad‐spectrum antimicrobial agents, such as piperacillin–tazobactam, carbapenems, ceftriaxone, and metronidazole, in addition to vancomycin or linezolid. For confirmed Group A streptococcal infections, clindamycin, and penicillin are recommended for 10–14 days [35].

In the present case, antimicrobial selection, dosage, and treatment duration were carefully adjusted due to the patient's chronic renal failure requiring dialysis, based on consultation with nephrologists, culture results, and previous literature [36]. Dose adjustment was carefully performed to avoid drug accumulation while maintaining therapeutic efficacy.

Although repeated debridement was performed and laboratory parameters gradually improved, necrosis continued to expand in the dorsal larynx and submandibular regions. Because the infection failed to resolve despite multiple debridements, surgical removal of the left submandibular gland was performed as a potential source of persistent infection. Histopathological examination revealed no evidence of active infection within the salivary gland parenchyma itself; however, inflammatory infiltration was present in the surrounding capsule. Following gland removal, inflammation gradually subsided, serum procalcitonin levels normalized, and further progression of necrosis was halted. This clinical course suggests that removal of the submandibular gland containing a suspected residual infectious focus may have contributed to a turning point in infection control. Taken together, these findings suggest that an initial pharyngeal infection may have extended to the capsule of the left submandibular gland, which could have acted as a persistent infectious focus contributing to the expansion of necrotic lesions.

This case emphasizes the importance of reassessing the infectious focus when cervical NF fails to respond to repeated debridement.

To the best of our knowledge, there have been no previous reports describing successful control of cervical NF through submandibular gland resection as a therapeutic strategy.

## Author Contributions

**Hiroyuki Makihara:** conceptualization, investigation, writing – original draft. **Shoichiro Kitajima:** investigation, resources, writing – review and editing. **Sanae Maeda:** supervision, writing – review and editing. **Susumu Mizuno:** supervision, writing – review and editing.

## Funding

The authors have nothing to report.

## Ethics Statement

Ethics approval was not required for this case report in accordance with local institutional guidelines.

## Consent

Written informed consent could not be obtained because the patient was deceased. All identifying information has been removed, and this case report has been anonymized in accordance with journal policies.

## Conflicts of Interest

The authors declare no conflicts of interest.

## Data Availability

Data sharing is not applicable to this article as no datasets were generated or analyzed during the current study.
